# Improving the accuracy of self-reported height and weight in surveys: an experimental study

**DOI:** 10.1186/s12874-022-01690-x

**Published:** 2022-09-19

**Authors:** Nina Van Dyke, Eric J. Drinkwater, Jerome N. Rachele

**Affiliations:** 1 Mitchell Institute, Victoria University, 300 Queen St, Melbourne, Australia; 2 The Social Research Centre, 5/350 Queen St, Melbourne, Victoria Australia; 3 Centre for Sport Research, School of Exercise & Nutrition Sciences, Deakin University, Geelong, VIC Australia; 4 School of Allied Health, Exercise and Sports Sciences, Charles Sturt University, Bathurst, New South Wales Australia; 5 College of Health and Biomedicine, Victoria University, Melbourne, Australia; 6 Institute for Health and Sport, Victoria University, Melbourne, Australia

**Keywords:** Body mass index, Self-report, Height, Weight, Surveys, Question-wording, Sensitive, Priming, Framing

## Abstract

**Background:**

Many studies rely on self-reported height and weight. While a substantial body of literature exists on misreporting of height and weight, little exists on improving accuracy. The aim of this study was to determine, using an experimental design and a comparative approach, whether the accuracy of self-reported height and weight data can be increased by improving how these questions are asked in surveys, drawing on the relevant evidence from the psychology and survey research literatures.

**Methods:**

Two surveys from two separate studies were used to test our hypotheses (Science Survey, *n* = 1,200; Eating Behaviours Survey, *n* = 200). Participants were randomly assigned to one of six conditions, four of which were designed to improve the accuracy of the self-reported height and weight data (“preamble”), and two of which served as the control conditions ( “no preamble”). Four hypotheses were tested: (H1) survey participants read a preamble prior to being asked their height and weight will report lower heights and higher weights than those not read a preamble; (H2) the impact of question-wording (i.e., preamble vs. no preamble) on self-reported weight will be greater for participants with higher BMIs; (H3) the impact of question-wording on height will be greater for older participants; (H4) either version of the weight question – standard or “weight-specific”—may result in participants reporting more accurate self-reported weight. One-way MANOVA was conducted to test Hypothesis 1; two-way analysis of variance were conducted to test Hypothesis 2; moderation analysis was used to test Hypothesis 3; independent samples t-test was conducted to test Hypothesis 4.

**Results:**

None of the hypotheses was supported.

**Conclusions:**

This paper provides an important starting point from which to inform further work exploring how question wording can improve self-reported measurement of height and weight. Future research should explore how question preambles may or may not operationalise hypothesised underlying mechanisms, the sensitivity or intrusiveness of height and weight questions, individual beliefs about one’s height and weight, and survey context.

## Background

Obesity is an important risk factor for a wide range of chronic diseases [28, 48, 69]. Despite research demonstrating the limitations of the use of body mass index (BMI) as a measure of body fatness [7, 44, 52, 53, 57], BMI continues to be used for clinical diagnoses [1, 8, 46] and to estimate population rates of overweight and obesity [2, 11, 29], with higher BMI associated with increased risk of obesity-related comorbidities and increased morbidity and mortality [16, 45, 71]. BMI is calculated by dividing a person’s weight in kilograms by their height in metres squared. A BMI of less than 18.5 is considered underweight, between 25 and 30 is categorized as overweight, and over 30 is considered obese [73].

Ideally, height and weight are measured by a clinician, using calibrated instruments such as a stadiometer for height and weighing scales for weight [19]. However, self-report measures are often used in large population health studies due to limitations in funding and resources [67, 70]. Research comparing self-reported height and weight data with clinical data generally finds discrepancies between the two sets of measurements, with certain groups of people over-reporting height and/or under-reporting weight [12, 23]. The result can be underestimation of BMI [26, 43] and misclassification of individuals as “underweight”, “normal weight”, “overweight” and “obese” [32], leading to lower estimates of obesity prevalence as well as greater random error [23]. Formulas designed to correct for this error have been only partly successful [3, 26, 46].

Given this reliance on self-report to calculate BMI, it is important to explore ways to gather more accurate data using this approach. One possibility largely ignored in the public health literature is to improve the way the questions about height and weight are asked in surveys. It has long been known in the survey research literature that how questions are asked can have a significant impact on responses [34, 55]. It therefore seems plausible that the accuracy of self-reported height and weight could be improved by asking the questions differently.

The primary aim of this study was to determine, using an experimental design, whether the accuracy of self-reported height and weight data can be increased by improving how these questions are asked in surveys. The findings will contribute to the evidence base on understanding self-reporting bias, and help integrate the literatures that currently exists somewhat separately in the psychological and survey research disciplines.

### Accuracy of self-reported BMI

Studies comparing measured and self-reported BMI find that, although the correlations between the two measures are generally high [13, 32, 47], there is a bias towards overreporting of height and underreporting of weight, resulting in an underreporting of BMI [23, 43] and subsequent misclassification of BMI categories among participants. This systematic error results in misclassification bias, of which there are two types: differential and non-differential. Differential misclassification is related to other study variables whereas non-differential misclassification is not (Rothman [54]:133). Non-differential misclassification is less likely to bias estimates, and tends to produce estimates that are “diluted” or closer to the null. This means that if there is no effect to begin with, non-differential misclassification is unlikely to bias the effect estimate (Rothman [54]:134). Biases from differential misclassification are less predictable, and can either exacerbate or underestimate an effect (Rothman [54]:134). The issue of misclassification bias is particularly pertinent for studies measuring self-reported height and weight: studies in which subgroups have an equal chance of misclassification of BMI categories have more predictable bias, and are less likely to be biased overall.

Existing research suggests that individuals with higher BMIs tend to underreport weight [32, 43, 63, 72], whereas older people tend to overestimate height [1, 64]. Thus, misclassification appears to be differential rather than non-differential [51]. Conclusions regarding the impact of this bias range from slight to significant [17, 20, 22, 24, 38, 47, 60]. Nevertheless, all agree that more accurate data is preferable.

### Explanations for this misreporting

To improve the accuracy of self-reported height and weight data, it is necessary to understand why these data are misreported. Whereas the psychological literature has mostly focused on the reporting of traits and attitudes, and the survey literature has emphasised the reporting of behaviours, it appears that similar processes lead to both types of misrepresentation [66].

The most commonly proffered explanation from both the psychology and survey methodology literature is social desirability [40]. This theory argues that people have a strong desire for others to see them in a positive light. In cultures that favour lower weight and greater height, people may report being taller and weighing less than their actual measurements to promote a more positive picture of themselves to others, such as a survey interviewer [39]. A recent study supporting this theory found that women’s social desirability score was significantly correlated with the discrepancy between self-reported and measured body weights after adjusting for their actual weight [41].

This distorted self-presentation may constitute either a “deliberately deceptive act” (i.e. impression management) or simply a “self-serving estimation error” (i.e. self-deception) [15, 49, 68]. DeAndrea et al. (2012) argue that one may distinguish between the two possibilities by establishing whether there is the presence of “ground truth” – i.e., knowledge of one’s true height and weight. In other words, if someone knows their actual height and weight, any reported distortion of these data is deliberate, whereas if they are unsure of their actual height and weight, or at least have convinced themselves that they are unsure of their actual height and weight, they may simply report data favourably. This theory suggests that, if one could either determine or enhance “ground truth”, accurate reporting of height and weight would be enhanced.

If the theory of social desirability is correct as applied to the self-reporting of height and weight, and people misreport their height and weight in order to influence an interviewer to think better of them, then one solution to this data bias problem would be to remove the influence of interviewers and instead conduct the survey using an anonymous mode, such as online or mail, rather than over the telephone or face-to-face. A considerable body of research, however, finds that in many cases more socially desirable responses are provided to survey questions even when there is no one asking the questions, thus casting doubt on this theory as the sole explanation for the misreporting of height and weight [25, 36]. Krueuter et al (2008), for example, found no differences in responses between interviewer- and self-administered modes for a set of five normative behaviours, including receiving academic honours and donating money to the university. Research by the Pew Research Center [35] found little difference in the reported frequency of church attendance by participants assigned randomly to a telephone interview or a web survey.

Another possible explanation for bias in self-reported height and weight is based on Identity theory, which concerns what people value and how people view themselves [61]. Rather than providing survey responses to convince the interviewer that they are a worthy person, survey participants may instead be expressing their self-identity as a worthy (i.e. a slightly taller and lighter) person. The participant sees themselves, or wants to see themselves, as healthy, active, and attractive, and thus responds to the height and weight questions in a way that more closely accords with this self-view. If someone values being fit and attractive, and views themself as being fit and attractive, they may underreport their true weight and/or overreport their true height as a low-cost opportunity to enact their identity [4]. Brenner & DeLamater ([4]:337) posit that, rather than being motivated solely by concerns regarding self-presentation, “*the respondent pragmatically reinterprets the question to be one about identity rather than behavior, a process influenced by a desire for consistency between the ideal self and the actual self. This pragmatic interpretation of the survey question encourages the respondent to answer in a way that affirms strongly valued identities.*” Identity theory, unlike social desirability theory, does not predict that responses to socially desirable questions will be more biased with non-anonymous survey modes (i.e. when another person is asking the questions), but instead predicts greater bias when self-identity does not accord closely with reality. Thus, conventional direct survey questions can prompt the participant to reflect not only on the actual self, but also on their ideal self [31].

### Impact of question wording on responses to sensitive questions

It is clear from the survey research literature that how survey questions are asked can have an impact on responses. This is particularly true for “sensitive” questions, such as illicit drug use, abortion, and sexual behavior, and “intrusive” questions such as household income, although what is considered sensitive or intrusive likely differs by demographic group, cultural background, [33] and individual [66].

There is evidence that specifically asking participants to provide accurate information, sometimes referred to as a *priming procedure*, improves accuracy of sensitive or intrusive survey questions [66]. Another promising approach to improving the accuracy of self-reported height and weight is by providing additional assurances regarding the *confidentiality* of the data, which has been shown to reduce misreporting [58]. Although most surveys provide such assurances at the start of the survey, or as part of the informed consent process, additional reassurance prior to asking the height and weight questions may improve reporting.

Finally, *framing effects* may be important [6]. Framing refers to the process by which people perceive and conceptualise an issue. Framing effects occur when changes in the presentation of an issue produce changes of opinion [9]. Two subtypes of framing effects are *wording* and *context* effects. Context effects refer to influence on survey responses by the context in which a question is asked. Wording effects refer to the language used to ask the question. These effects have been observed on an array of issues [18, 21, 50]. Although normally discussed in relation to attitudes, framing effects may also be important for other types of survey responses, such as self-reporting of height and weight. Little research, however, has examined its impact on these types of questions.

Magelssen et al. [42], for example, examined the impact of context and wording on support for assisted dying in Norway, by randomly assigning participants to different versions of the same questions. In one version, participants were simply asked whether they agreed or disagreed that physician-assisted suicide should be allowed for persons who have a terminal illness with short life expectancy. The second version added additional information that included an example of a particular patient who ‘is in great pain’, careful consideration by a doctor, and the choice of the patient to ‘avoid great suffering.’ Whereas the first version asks about ‘physician-assisted suicide’ and ‘euthanasia’, the second version uses the phrase, ‘a lethal drug dose that the patient can choose to take to avoid great suffering’. The result is significantly greater support for assisted dying by participants assigned to version 2. Another example of wording effects in the area of economic attitudes finds that expectations and perceptions regarding future inflation rates were lower and less variable when participants were asked about “inflation” as opposed to “prices in general” or “prices you pay” [14]. These effects of context and wording, however, do not always hold. Singer and Couper [59], for example, conducted an experiment in which they randomly assigned participants to questions about attitudes toward prenatal testing and abortion framed either in terms of “baby” or “fetus”, with the expectation that support would be higher for those assigned to the second version. They found, however, no significant differences by question wording for abortion preferences and small but significant differences for prenatal testing. They did, however, find that question wording made substantial differences in the responses of some demographic subgroups. It may be that attitudes towards abortion are so strongly held by many that framing effects have little impact.

Finally, the presence of an *authoritative citation*, where the question is asked with the addition of an authoritative statement supporting it, has also been shown to affect survey responses – again, mostly on attitude questions [10]. Cocco & Tuzzi [10], in an Italian study examining the impact of question-wording and context on attitudes towards homosexual behaviour and a possible law against homophobia, found more negative responses with the addition of the following statement: “Silvio Berlusconi has stated that it is better to appreciate beautiful girls than to be gay.” One may argue about the “authoritativeness” of such a statement; nevertheless, the point holds that the statement is attached to a person of authority.

The aim of this study was to determine, using an experimental design, whether the accuracy of self-reported height and weight data can be increased by improving how these questions are asked in surveys, drawing on the relevant evidence from the psychology and survey research literatures. Four hypotheses are tested. These hypotheses are stated in the Methods section, below.

## Methods

Two surveys from two separate studies were used to test our hypotheses. Ethics approval for Study 1 (“Science Survey”) was provided by the Australian National University Human Research Ethics Committee. Ethics approval for Study 2 (“Eating Behaviours (EB) Survey”) was provided by the Charles Sturt University Human Research Ethics Committee. All methods were performed in accordance with guidelines and regulations set out by the above institutions.

### Participants and procedure

The Science Survey consisted of a Random Digit Dialling (RDD) Computer Assisted Telephone Interview (CATI) survey of 1200 Australian adults (aged 18 +) across Australia. The EB Survey consisted of an RDD CATI survey of 200 non-metropolitan Australian adults (aged 18 +). The participation rate (AAPOR 2016) for the Science survey was 43.2%. Of the 5,637 telephone numbers dialled, 1,065 were unusable (e.g. disconnected; not a residential number), for 1,371 there was no contact (e.g. no answer; answering machine; engaged), and 426 were deemed out of scope (e.g. non-English speaking; no one age 18 + in household). Of the 2776 telephone numbers considered in scope, 1200 interviews were completed. The participation rate for the EB survey was 34.7%. Of the 2,867 telephone numbers dialled, 1,524 were unusable, for 522 there was no contact, and 79 were deemed out of scope. Of the 742 telephone numbers considered in scope, 200 interviews were completed.

Informed consent was obtained from each participant before starting the survey. No incentive was provided for participation. Both surveys were conducted by the Social Research Centre, a social research company.

Key demographics of the two samples are presented in Table 1. Given that the population of the EB Survey was non-metropolitan Australians whereas the population of the Science Survey was all Australians, it is not surprising that Science Survey participants were more highly educated, had higher incomes, and had lower BMIs (AIHW 2017) as compared with the EB Survey participants; the Science Survey sample also had a more even mix of men and women.

**Table 1:** Survey sample characteristics

	**Science Survey (** ***n*** ** = 1200)**		**EB Survey** **(** ***n*** ** = 200)**	
**Variable**	**n**	**%**	**n**	**%**
***Age***	**1170**	**97.5**	**200**	**100.0**
18–34 years	200	17.1	35	17.5
35–54 years	467	39.9	92	46.0
55 + years	503	43.0	73	36.5
*Mean*	*51.03*		*43.58*	
*Standard deviation*	*16.30*		*17.88*	
***Gender***	**1200**	**100.0**	**200**	**100.0**
Female	609	50.8	114	57.0
Male	591	49.2	86	43.0
***Location***	**1200**	**100.0**	**200**	**100.0**
Metro	n/a	n/a	0	0
Non-metro	n/a	n/a	200	100
Australian Capital Territory	100	8.3	0	0
New South Wales	234	19.5	53	26.5
Northern Territory	100	8.3	11	5.5
Queensland	212	17.7	49	24.5
South Australia	127	10.6	24	12.0
Tasmania	100	8.3	16	8.0
Victoria	191	15.9	27	13.5
Western Australia	136	11.3	20	10.0
***Highest education***	**1185**	**98.8**	**193**	**87.7**
Completed less than Year 12	282	23.8	63	32.6
Completed Year 12	207	17.5	34	17.6
Completed TAFE or other certificate	308	26.0	47	24.5
Completed university or higher	388	32.7	51	26.6
***Household income***	**942**	**78.5**	**186**	**93.0**
< $80,000	537	57.0	116	62.4
> = $80,000	405	43.0	70	37.6
***BMI***	**1080**	**90.0**	**192**	**96.0**
Underweight (< 18.5)	24	2.3	3	1.6
Normal (18.5–25)	482	40.2	65	33.9
Overweight (25–30)	377	31.4	78	40.6
Obese (> 30)	197	16.4	46	24.0
*Mean*	*26.21*		*27.22*	
*Standard deviation*	*5.04*		*5.14*	

### Measures

The focus of the Science Survey was on public attitudes towards science. The questions relevant to this study comprised a module in the second part of the survey. The height and weight questions were asked after the science attitude questions and a general health question, but before the demographic questions. The focus of the EB Survey was on eating behaviours and physical and mental health. The height and weight questions were asked after a series of questions about eating behaviours, attentiveness to messages about dieting, health conditions, and smoking behaviour.

In both surveys, participants were randomly assigned to one of three versions of a preamble to the height and weight questions. They were then independently randomly assigned to one of two versions of the weight question. Therefore, in each survey, each participant was asked one of six combinations of height and weight questions (see Table 2). The height question was always asked before the weight question.

**Table 2:** Height and weight questions

Version	Height question			Weight question		n
**Science Survey**						
	*Science_a. (No preamble)*	*Science_b. I’m now going to ask you your height and weight. It’s very important that we get as accurate data on these questions as possible*	*Science_c. I’m now going to ask you your height and weight. Research shows that people tend to overestimate their height and underestimate their weight. It’s very important that we get as accurate data on these questions as possible*	*Science_Wa. How much do you weigh? (ONLY IF QUERIED: ‘Without clothes or shoes’)*	*Science_Wb. How much do you weigh without clothes or shoes?*	
	*How tall are you without shoes?*	*Can you tell me, how tall are you without shoes?*	*Can you tell me, how tall are you without shoes?*			
Sci-1	x			x		172
Sci-2	x				x	190
Sci-3		x		x		224
Sci-4		x			x	217
Sci-5			x	x		200
Sci-6			x		x	171
**EB Survey**						
	*EB_a. (No preamble)*	*EB_b. I’m now going to ask you your height and weight. Please respond honestly. Our data rely on honest answers. Remember your name is not associated with your responses, so no one will know your height and weight*	*EB_c. I’m now going to ask you your height and weight. Research shows that people tend to OVERestimate their height and UNDERestimate their weight. It’s very important that we get as accurate data on these questions as possible*	*EB_Wa. How much do you weigh? (ONLY IF QUERIED: ‘Without clothes or shoes’)*	*EB_Wb. How much do you weigh without clothes or shoes?*	
	*How tall are you without shoes?*	*Can you tell me, how tall are you without shoes?*	*Can you tell me, how tall are you without shoes?*			
EB-1	x			x		37
EB-2	x				x	38
EB-3		x		x		34
EB-4		x			x	26
EB-5			x	x		29
EB-6			x		x	36

In the Science Survey, participants were randomly assigned to one of the following “preamble conditions”: (a) “no preamble” condition, in which they were simply asked how tall they are without shoes; (b) “accountability/priming” condition, in which the interviewer first stressed the importance of gathering accurate data on height and weight before asking the height question; or (c) “authoritarian citation/accountability/priming” condition, in which participants were also told that research shows that people tend to overestimate height and underestimate weight. In the EB Survey, participants were randomly assigned to one of the following “preamble conditions” (a) “no preamble” condition, identical to the Science Survey; (b) “context/priming/confidentiality” condition, in which the interviewer not only stressed the importance of accuracy in gathering height and weight data, but also reiterated participant anonymity; or (c) “context/priming/authoritarian citation” condition, which was similar to the Science Survey condition.

Following the height question, participants were again randomly assigned – this time to one of two weight questions. The “standard” version simply asked the participant to report their weight. Only if queried were they told this meant their weight without clothes or shoes. The “specific” version specified weight without clothes or shoes. Table 2 indicates the 12 (six for each study) combinations of height and weight questions posed to participants along with the number of participants in each condition.

### Hypotheses

Using an experimental design, this study tested whether the addition of preambles to the height and weight questions would result in more accurate self-reported height and weight responses. As we did not have data on participants’ actual height and weight, we used the comparative approach, whereby lower height and higher weight are assumed to be more accurate. The comparative approach is used when objective criterion (such as measured heights and weights of Australians and rural Australians) are lacking and when a known bias (i.e., underreporting of weight and overreporting of height) exists [37]. In addition, we tested hypotheses supported by the literature regarding the differential impact of improved question-wording on specific sub-population groups.

### The following hypotheses were tested

- **Hypothesis1: **Participants read a preamble (either Sci-3–4 (accountability + priming) or Sci-5–6 (accountability + priming + authoritarian citation; or EB-3–4 (context + priming + confidentiality) or EB-5–6 (context + priming + authoritarian citation)) prior to being asked the height and weight questions will report lower height and higher weight, on average, than those who were not read a preamble (Sci-1–2; EB-1–2).
- **Hypothesis 2:** The association between question-wording (i.e. preamble vs. no preamble) and self-reported weight will be greater for individuals with higher BMIs.
- **Hypothesis 3:** The association between question-wording and self-reported height will be greater for older participants.
- **Hypothesis 4:** We also test, but have no hypothesis regarding, whether the “standard” or “specific” weight questions (i.e. Sci-3–6 vs. Sci-1–2 and EB-3–6 vs. EB-1–2) will result in more accurate (i.e. higher) self-reporting of weight. On the one hand, we would predict that the “specific” version – “without shoes or clothes” – should result in more accurate weights because people weigh slightly less without clothes and shoes. On the other hand, the additional wording in the “specific” version may prime participants to report more accurate responses [15], which should result in higher self-reported weights.

### Statistical analyses

Sample sizes were based largely on practical considerations and thus no a priori calculation of sample sizes was conducted. For the Science survey, the relevant questions were added to an existing planned survey that required a sample size of 1200. The sample size (*n* = 200) for the EB survey was determined by budget. The variables, *height* and *age,* satisfied standard tests for normality and other statistical assumptions; *weight* and *BMI* were positively skewed. Parametric tests were employed as the use of non-parametric tests has several significant disadvantages and sample sizes were large enough that skewness does not make a substantial difference in the analysis (Tabachnick & Fidell [62]:80). All observed height and weight outliers were included in the analysis as the reported values were in a plausible range [27].

The two surveys were analysed separately as their populations were different (i.e., all Australians 18 + ; non-metropolitan Australians 18 +). The following tests were used to examine demographic differences between the six different question-version groups: chi-square test for independence (Gender); Krukal-Wallis test (Education; Household income); one-way analysis of variance (ANOVA) (Age). One-way multivariate analysis of variance (MANOVA) was conducted to test Hypothesis 1. Moderation analysis using the Hayes PROCESS macro was used to test Hypothesis 3. As BMI is calculated using weight (the dependent variable), moderation analysis was not appropriate for testing Hypothesis 2. Instead, two-way ANOVAs were conducted with question-wording and BMI categories (“normal weight”, “overweight”, “obese”) as factors, in order to examine the interaction between question-wording and BMI. As few participants were classified as “underweight” based on BMI (Science Survey: n = 24, 2.2%; EB Survey: n = 5, 2.4%), they were not included in this analysis. Independent samples t-test was conducted to test Hypothesis 4. All analyses were conducted using IBM SPSS Statistics for Windows, version 26 (IBM Corp., Armonk, N.Y., USA).

## Results

There were no differences in either survey among the six question-version groups on the following demographics: gender^1^ (Science survey: Chi-square (5, n = 1180) = 3.19, *p* = 0.67, *Cramer’s V* = 0.05; EB Survey: Chi-square (5, n = 200) = 9.33, *p* = 0.10, *Cramer’s V* = 0.22), education (Science survey: Chi-square (5, n = 1165) = 6.06, *p* = 0.300; EB Survey: Chi-square (5, n = 198) = 7.22, *p* = 0.205), or household income (Science survey: Chi-square (5, n = 927) = 4.30, *p* = 0.507; EB Survey: Chi-square (5, n = 185) = 8.56, *p* = 0.128). For age, there were no significant differences among the six question-version groups in the Science survey (*F* (5, 1144) = 0.23, *p* = 0.95), but a significant difference between EB-4 (mean = 53.5) and EB-5 (mean = 37.1) in the EB survey (*F* (5, 193) = 2.77, *p* = 0.02).

### Hypothesis 1: Survey participants read a preamble prior to being asked their height and weight will report lower heights and higher weights than those not read a preamble

As can be seen in Tables 3 and 4, there was no significant difference between the no preamble and preamble groups on self-reported height or weight. Therefore, Hypothesis 1 was not supported. Participants in the Science Survey read either the Sci-3 or Sci-4 (accountability; priming) or Sci-5 or Sci-6 (accountability; priming; authoritarian citation) preambles did not report lower heights and higher weights as compared with participants read no preamble (Sci 1 or Sci-2). Similarly, participants in the EB Survey read the EB-3 or EB-4 (context + priming + confidentiality) or EB-5 or EB-6 (context + priming + authoritarian citation) preambles did not report lower heights and higher weights as compared with participants given no preamble (EB-1 or EB-2).

**Table 3:**
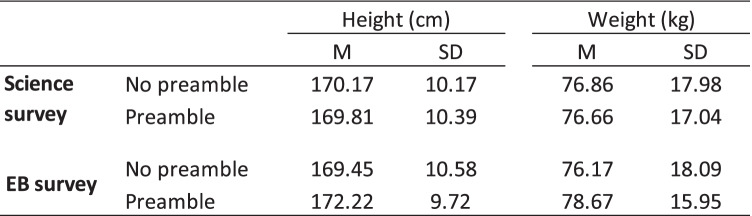
Mean scores and standard deviations for Height and Weight as a function of question-wording (preamble vs. no preamble)

**Table 4:**
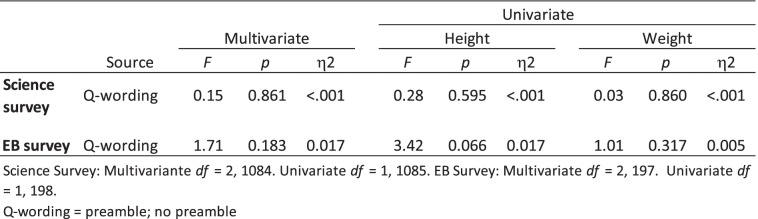
Multivariate and univariate analyses of variance for height and weight

A comparison of each of the preambles separately with the no preamble condition (i.e. no preamble vs. accountability + priming vs. accountability + priming + authoritarian citation in the Science survey, and no preamble vs. context + priming + confidentiality vs. context + priming + authoritarian citation) also resulted in no statistically significant differences, although we note that in the EB survey, mean weight for the context + priming + confidentiality condition was 5.4 kgs higher than for the no preamble condition (81.6 kg (SD = 17.35) vs. 76.2 kg (SD = 18.09)), and 5.1 kgs higher than for the context + priming + authoritarian citation condition (76.5 kg (SD = 14.57); (*F* (2, 197) = 2.02,* p* = 0.135*)*. Neither of the preambles in the Science Survey included a confidentiality statement.

### Hypothesis 2: The impact of question-wording (i.e., preamble vs. no preamble) on self-reported weight will be greater for participants with higher BMIs

The mean scores and standard deviations for weight as a function of BMI category are presented in Table 5. The interaction effects between question-wording and BMI category were not statistically significant (Science survey: *F* (2, 1057) = 0.65, *p* = 0.52. EB survey: *F* (2, 189) = 2.13, *p* = 0.122). Therefore, Hypothesis 2 was not supported.

**Table 5:**
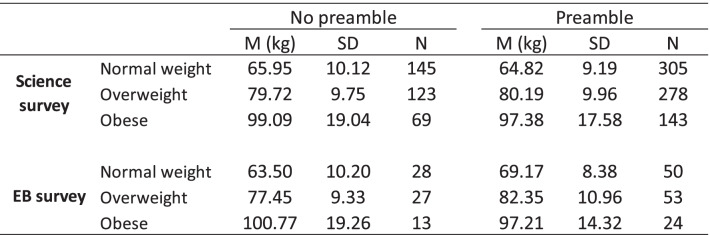
Mean scores and standard deviations for weight as a function of BMI category

### Hypothesis 3: The impact of question-wording on height will be greater for older participants

**Fig. 1:**
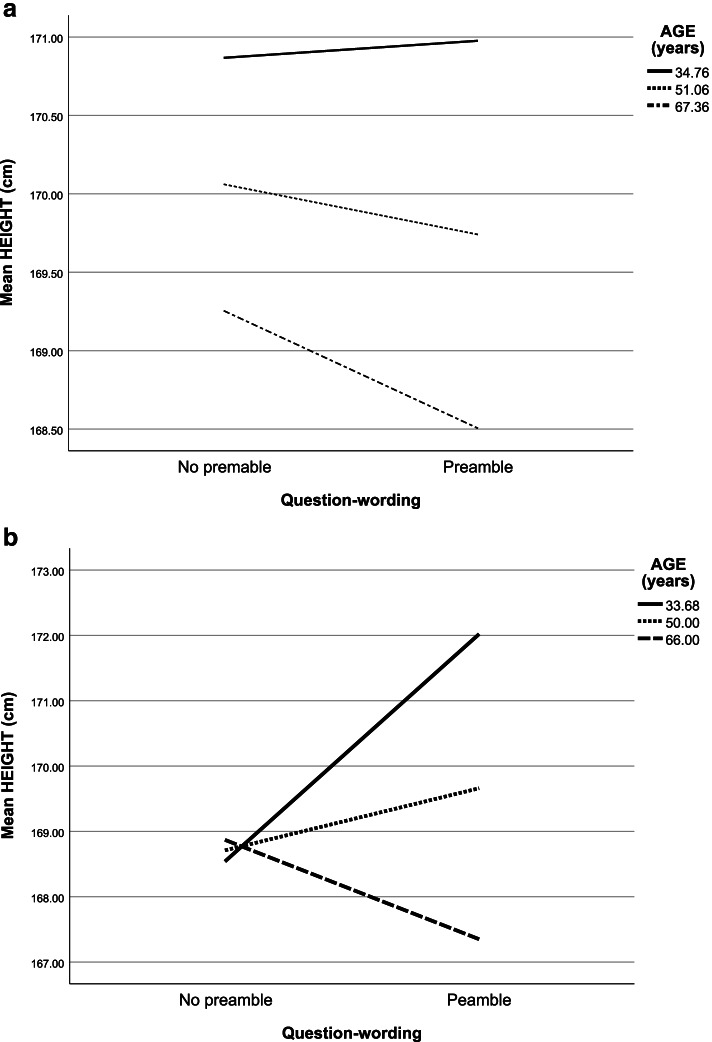
**a** Science Survey: simple slopes analysis of Age as a moderator of the relationship between question-wording and self-reported height. **b** EB Survey: simple slopes analysis of Age as a moderator of the relationship between question-wording and self-reported height

As shown in Table 6 and Figs. 1a and b, although slopes were in the expected directions, the interaction between question-wording (i.e. preamble vs. no preamble) and age was found to be not statistically significant. Therefore, hypothesis 3 was not supported.

**Table 6:**
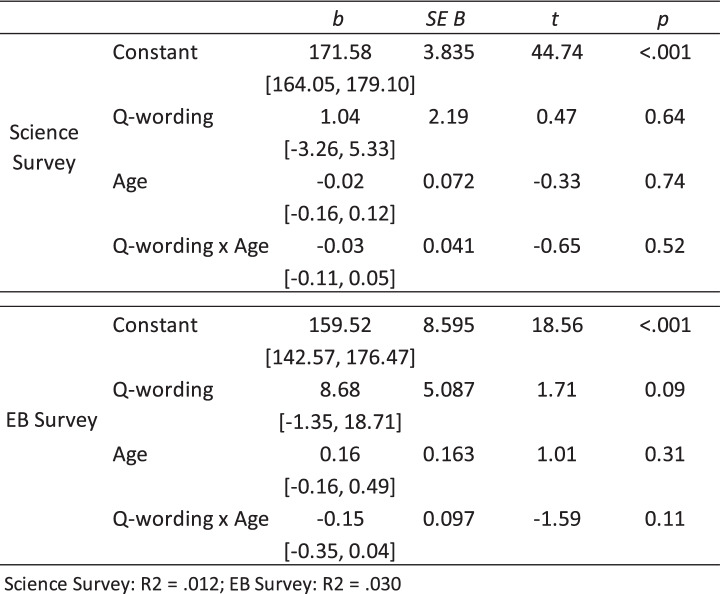
Age as a moderator between question-wording and self-reported height

### Hypothesis 4: Either version of the weight question – standard or “weight-specific”—may result in participants reporting more accurate self-reported weight.

As can be seen in Table 7, there was no significant difference between the standard and specific groups on self-reported weight. Therefore, Hypothesis 4 was not supported.

**Table 7:**
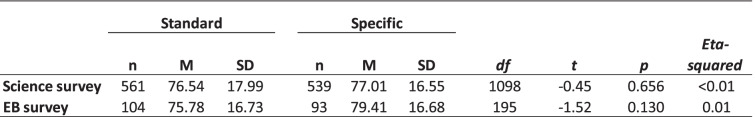
Differences in self-reported weight for standard vs. specified weight question

## Discussion and conclusions

No significant differences were observed in self-reported height or weight between survey participants who were read preambles to the height and weight questions designed to elicit more accurate responses (i.e. lower heights and higher weights), and those who were not. There was also no support for the hypothesis that the impact of question-wording on self-reported weight would be greater for those participants with higher BMIs or for the hypothesis that the impact of question-wording on self-reported height would be greater for older participants.

In designing the preambles, we focused on those mechanisms identified in the literature as improving the accuracy of responses to questions deemed sensitive or intrusive; namely, accountability; priming; assurances of confidentiality, framing, wording, and context; and authoritative citation. One possible explanation for our results is that the wording of the preambles did not correctly operationalise the hypothesised underlying mechanisms. For example, perhaps the phrase, “*Research shows that people tend to OVERestimate their height and UNDERestimate their weight”* did not correctly operationalise the mechanism of authoritative citation. It is also possible that participants didn’t pay much attention to the preamble and simply heard the questions about height and weight. This is more of an issue with web surveys, however [5], than when an interviewer is reading out the questions.

Another possibility is that the identified mechanisms don’t apply to questions about height or weight. Perhaps questions about height and weight are simply not sensitive or intrusive enough to be amenable to manipulation by question wording [65]. An examination of the data revealed that while just 0.3% of participants in the Science survey refused to answer a “neutral” question about whether they have a scale in their house, 2.3% refused to provide their weight and 1.2% refused to provide their height. This compares with 2.5% that refused to provide their age, and 10.7% that refused to provide their household income -- the latter question having notoriously high refusal rates [74]. These data suggest that questions about height and, in particular, weight, are arguably “sensitive”. In the EB survey, however, no one refused to provide their height and just 0.5% (1 participant) refused to provide their weight. This difference in response rates to these questions between the two surveys may have something to do with within-survey context – the EB survey was introduced as a survey about health and eating behaviours, and thus questions about height and weight were likely not unexpected. The Science survey, in contrast, was introduced to participants as a survey about attitudes towards science, with most questions on this topic, and thus questions about one’s height and weight may have been viewed as unexpected and therefore sensitive or intrusive. Alternatively, the lower refusal rates in the EB survey may be due to the different populations of the two surveys – non-metropolitan residents in the EB survey as compared with mostly metropolitan residents in the Science survey. The refusal rates for age (0%) and household income (0.3%) were also very low in the EB survey, although an additional 9.8% of participants said they “didn’t know” their household income, which may indicate soft refusals [30, 56].

Alternatively, perhaps beliefs about one’s height and weight are so firmly fixed – whether due to faulty memory (for example, what one weighed as a young adult as opposed to now) or a strong identity attachment to being taller and thinner than one actually is [4] – that promptings designed to trigger the identified mechanisms simply fail [59]. However, evidence that people who strongly suspected they would be weighed and measured following questions about their height and weight were less likely to bias their self-reports [72] suggests this may not be the case. Instead, *accountability* may be key to counteracting this distortion of self-presentation. According to impression management theory, it may do more damage to one’s impression management to be caught lying about one’s height and weight than to be seen as shorter and heavier than is societally desirable [15]. The survey research literature refers to this phenomenon as the “bogus pipeline” [27, 66]. Großschadl et al. (2012), for instance, posited that an explanation for their finding of fewer discrepancies between self-reported and actual height and weight measurements for women and older people than those found in most other studies was that participants completed the survey as part of a health check, and thus likely knew that they would also have their height and weight measured. It is also possible that people who volunteer for a health check are more aware of their actual height and weight, and therefore have greater “ground truth.” Although several of the preambles attempted to trigger “accountability” by stressing the importance of gathering accurate data, this is surely a weaker prod than the “threat” of being weighed and measured. Future studies may want to consider asking participants (who own working scales and/or tape measures) to weigh and measure themselves and report this data, to see if this increases accuracy. This would also help establish *ground truth* (i.e. their actual measurements), and thus help determine whether biased reporting is a “deliberately deceptive act” or simply a “self-serving estimation error” [15].”

Finally, it is possibly that the broader context of the survey plays a role. In the Science survey, where most of the survey questions asked about attitudes towards science, none of the group differences by preamble condition was close to statistically significant. In the EB survey, however, which focused on health and eating behaviours, most of the differences were in the expected directions and several approached statistical significance despite the small sample size.

Limitations of this study, in addition to the small sample size of the EB survey and use of the comparative approach in lieu of comparison with measured data, include that the EB survey was limited to non-metropolitan residents. It is possible that rural Australian are impacted by question-wording about height and weight differently than metropolitan residents. Another limitation is that the surveys used for these analyses were designed to serve a number of purposes, and therefore some of the questions were not ideally designed to answer the research questions posed in this study. In particular, each of the preambles combines mechanisms purported to impact responses, rather than testing each individually, such that it is impossible to disentangle the impact of each.

Nevertheless, and despite a lack of support for any of the four hypotheses, we believe that this paper makes an important contribution to the literature. From a population health perspective, it is important that self-reported height and weight be as accurate as possible and that we continue to seek ways to achieve this. Despite evidence from the survey research literature that question-wording can have a significant impact on responses, to date little research has examined whether the accuracy of self-reported height and weight data can be improved by asking these questions differently. This study aimed to do this, drawing on both the survey research evidence on question-wording and the psychological literature on self-report bias. We believe that this study makes an important contribution to the evidence regarding self-report bias, as well as discussing some promising avenues for future research on this topic.

In particular, we recommend conducting an adequately powered study focused on health that tests both single mechanisms as well as combinations of mechanisms, in order to systematically determine whether and when question-wording can improve the accuracy of self-reported height and weight. Specifically, we believe it is worth further exploring the accountability mechanism by incorporating the possibility of actual height and weight measurements. We also propose further testing of the confidentiality mechanism, which was included in only one of the EB survey preambles. Although not statistically significant, the mean self-reported weight for the context + priming + confidentiality condition was 5.4 kgs higher than for the no preamble condition, and 5.1 kgs higher than for the context + priming + authoritarian citation. Other recommendations for future research are to include measures of social desirability [41] and identity attachment, to better understand their role in the self-reporting of height and weight. It would also be useful to explore other theoretical explanations – beyond social desirability and identity theory – for the misreporting of height and weight, and how these might be addressed. Finally, qualitative research would be useful to better understand the extent to which people associate being taller and/or thinner as an ideal and how this may impact on the self-reporting of height and weight.

## Data Availability

The “Science Survey” dataset analysed during the current study is available in the Australian Data Archive repository, https://dataverse.ada.edu.au/dataverse/ada The “EB Survey” dataset analysed during the current study is available from the corresponding author on reasonable request.

## Abbreviations

BMI: Body mass index
Study 1: Science survey
Study 2: Eating behaviours survey
EB: Eating behaviours
RDD: Random digit dialling
CATI: Computer assisted telephone interview
ANOVA: Analysis of variance
MANOVA: Multivariate analysis of variance

## Footnotes

1 The gender item occurred towards the end of the survey. Survey interviewers were instructed to assign a gender; if they were unsure of the respondent’s gender, they were instructed to ask ‘What is your gender?” with coded response options: Male; Female; Other/transgendered/intersex, etc.
